# Tuberculosis among Migrant Populations in Sicily: A Field Report

**DOI:** 10.1155/2021/7856347

**Published:** 2021-03-30

**Authors:** Tullio Prestileo, Giuseppe Pipitone, Adriana Sanfilippo, Antonio Ficalora, Giuseppe Natoli, Salvatore Corrao, Team I. Ta. C. A. (Immigrant Take Care Advocacy) Team

**Affiliations:** ^1^Infectious Disease Unit & Centre for Migration and Health, ARNAS Civico Di Cristina Benfratelli Hospital, Palermo, Italy; ^2^StopTB Section of Sicily, Palermo, Italy; ^3^Systemic and Immune-Depression Associated Infectious Diseases Unit, National Institute for Infectious Diseases Lazzaro Spallanzani IRCCS, Rome, Italy; ^4^Department of Internal Medicine, ARNAS Civico Di Gristina Benfratelli Hospital, Palermo, Italy; ^5^PROMISE Department, University of Palermo, Palermo, Italy; ^6^ANLAIDS (Associazione Nazionale per la Lotta all'AIDS) Sicilia, Palermo, Italy

## Abstract

**Background:**

In the EU, tuberculosis (TB) mainly affects vulnerable people, including migrants. From 2014 to 2017, we have estimated the frequency of both tuberculosis and latent tuberculosis infection (LTBI) among the migrant population hosted in 41 reception centers in western Sicily (ITaCA network).

**Materials and Methods:**

All migrants were consecutively recruited for the screening of TB infection with physical examination and TST in 1,020 migrants and with IGRA in the others 2,690. The screening was carried out 4–8 weeks after landing in Sicily. For all migrants with a positive screening test, chest X-ray and smear examination were performed. LTBI was defined by positivity of TST or IGRA with negative X-ray chest, clinical, and smear examination. Active TB was defined by radiological and/or clinical and/or sputum positivity in a patient with a TST or IGRA positivity.

**Results:**

We evaluated a total of 3,710 migrants, of which 89% came from Sub-Saharan countries; 2,811 were males, 899 were females, with a median age of 22 years (IQR: 18–25). TB infection was diagnosed in 501 persons (13.5%) of which 440 (11.8%) had LTBI and 61 had active TB (1.6%): 1 had lymph node TB, 1 had intestinal TB, and 59 had pulmonary TB (38 sputum smear positive TB; no drug-resistant TB were observed).

**Conclusions:**

TB screening is critical to early diagnosis and treatment.

## 1. Background

Globally, by the end of 2017, more than 68 million people are estimated to be forcibly displaced[1, 2]. Specific attention has been paid to migrants fleeing to Europe as thousands have lost their lives during their attempt to cross the Mediterranean Sea. Because of its geographical proximity to Africa, Italy is a major point of entry for migrants to Europe and, in 2017, received about 120,000 migrants [3].

Migrants arriving to the European Union (EU) experience worse health determinants compared to host populations and often present late for care upon arrival in the EU. Migrants are more exposed and vulnerable to the acquisition of many communicable diseases [4]. On the other hand, the “healthy migrant effect” sees individuals who are young and in good physical condition during the journey across the harsh conditions in the hope of a better future in Europe. Although being in good clinical condition, these migrants may be carriers of asymptomatic chronic infections such as tubercular infection and others communicable diseases, and their subsequent status may be unknown because they come from countries with weak health systems or because prolonged conflicts and poverty have hindered access to quality healthcare, such as screening and vaccination [5].

Short-term cost savings linked to limiting migrants' access to preventive care, early diagnosis, and care for communicable and noncommunicable diseases (NCDs) are often soon lost due to the cost of provision of emergency care and treatment to individuals diagnosed late [6].

The exact number of infections among migrants and knowledge about the associated risk factors are lacking [7]. Estimates are required of migrant groups that are most affected and, therefore, would benefit the most from targeted screening programs, early detection, and treatment, to achieve the World Health Organization (WHO) goal to reduce 80% drop of new TB cases and to reduce the number of TB deaths [8].

Migrants arrived in Italy mainly through the Mediterranean routes. The majority of them landed in Sicily, mostly in western Sicily, due to its proximity to the North African coast.

We evaluate 3,710 migrants, a small part of the total number of migrants landed in western Sicily (48,043 migrants) in the observational period (2014–2017): the majority of migrants, through Mediterranean routes, arrived in the Mediterranean Sea and were rescued by the international SAR (Safe and Rescue) project. Most of them were transported to the Sicilian ports, about half of them in western Sicily (48,043), and then to the identification center.

Many other migrants landed by themselves in the coast of Sicily (and islands around it such as Lampedusa and Pantelleria) and also Malta and Cyprus. We do not know the exact number of them because they often were not identified by the SAR project and by national officers. A small part of these irregular migrants was stopped by a police officer and were transported to an identification center.

On the other hand, the migrants landed with the SAR project can be more easily identified and provided access to medical care, but in the first 4 weeks, they were relocated in many centers through a process of personal data registration and redistribution the last month (almost 1 or 2 years). During this period, many migrants escape from the center. So, we can only estimate the total number of migrants landed in Italy. We have, therefore, chosen to analyze a part of migrants.

41 centers in western Sicily were selected (which were part of the National ITaCa Project), to which migrants had been transferred. Once or twice in a week, an ITaCa team (a doctor, a nurse, and a cultural mediator) went to the center to perform medical examination, TST/IGRA, and chest X-ray. All patients of the 41 centers were screened, 4 to 8 weeks after arrival in Italy. When an ITaCa team member cannot go to the center to read the TST, every center had a doctor who helped in this task.

The aim of our study was to estimate the frequency of latent tuberculosis infection (LTBI) among the migrant population. To achieve this, we followed the WHO recommendation that suggest systematic testing for and treatment of LTBI for immigrants from countries with high TB burden [9]. The population we observed was almost entirely composed of migrants from countries with high TB endemic, except for Mali and other countries.

## 2. Population Sample and Research Methods

From 2014 to 2017, 48,043 migrants landed in western Sicily and 49.8% remained in the reception centers in the same territory. We evaluated only the migrants transported to the ports of Lampedusa, Palermo, Trapani, and Porto Empedocle.

At arrival, the migrants were triaged to identify their health status: migrant people with signs and/or symptoms of communicable disease and NCDs that need hospitalization were transported to health facilities for diagnosis and treatment and were excluded. The other migrants were transported to the Sicilian ports for the identification and then to a center for migrants in the whole Italian territory. We evaluate only the migrants hosted in the 41 identification centers in the provinces of Agrigento, Palermo, and Trapani. Among the 48,043 migrants landed in western Sicily between 2014 and 2017, we evaluate 3,710 migrants hosted in 41 Sicilian reception centers who cooperatedto the Immigrant Take Care Advocacy (ITaCA) network team with the Infectious Diseases Unit and Center for Migration and Health of the ARNAS-Civico Hospital of Palermo.

The procedures followed were in accordance with the ethical standards and with the Helsinki Declaration of the World Medical Association.

During our study, we have collected data on sociodemographic variables and migration history, 4 to 8 weeks after their arrival in the reception centers. Medical procedures were explained in English, French, or Arabic, and written or verbal informed consent was obtained.

Migrants were supported by a cultural mediator throughout the process, and medical procedures were explained in their native language. Following the completion of the survey, migrants were informed about the importance of early screening for TB infection.

**Figure 1:**
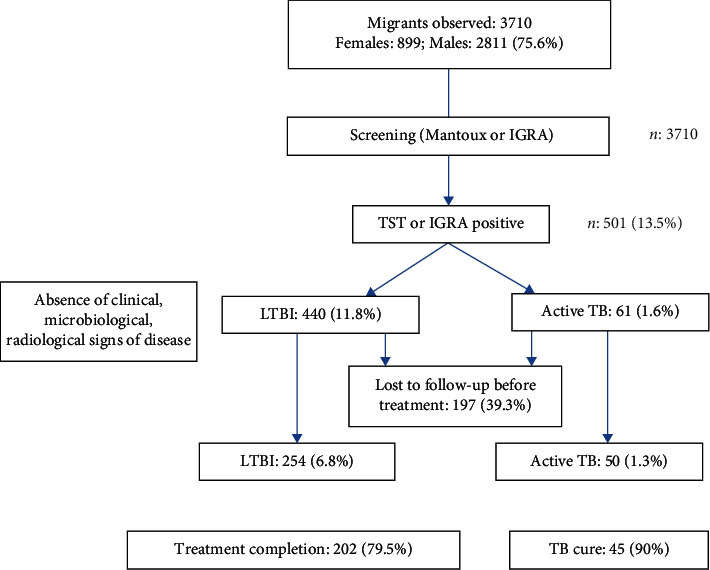
Flow chart and methodology.

Following the National Guideline for the Migrant's Health, updated in 2017, we perform the screening using the Mantoux test (Tubercolin Skin Test, TST) or IGRA (Interferon Gamma Release Assay) test. IGRA was performed if the migrant could not be re-evaluated within 72 hours. We assumed a TST positivity of 15 mm or more or 10 mm if migrants come from a highly endemic country (TB incidence >100/100.000) [10]. Moreover, all migrants were screened for symptoms. So, among migrants with a positive TST/IGRA and/or the symptomatic ones, we subsequently performed an X-ray chest and sputum examination for Bacillus of Koch (BK) research with PCR and smear examination (Figure 1). In such way, we try to estimate the prevalence of active TB.

We used the following definition to define the patients with TB infection:

- TB case: based on radiological, clinical, and microbiological evaluation, associated with TST or IGRA positivity
- LTBI: TST or IGRA positivity in the absence of clinical, biohumoral, microbiological, and radiological signs of disease
- Pulmonary active TB: it was defined by radiological and/or clinical and/or sputum positivity in a patient with a TST or IGRA positivity

Data were reported as percentages for categorical variables and as means (95% confidence intervals) and median (interquartile range) for quantitative variables. Stata (StataCorp. 2016. Stata Statistical Software: Release 14.1. College Station, TX: StataCorp LP) was used for database management and analysis.

IGRA test: LIAISON QuantiFERON-TB Gold Plus Solution. Four Blood Collection Tubes (Nil, TB1, TB2, and Mitogen): DiaSorin, Saluggia (VC), Italy; PCR test: COBAS AMPLICOR; and MTB PCR assay, Roche.

## 3. Results

**Figure 2:**
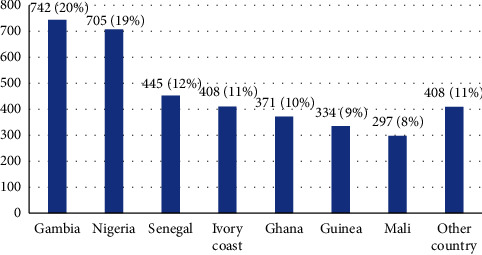
Migrants' country of origin (total number: 3710).

From 2014 to 2017, 3,710 African migrants were observed, of whom 75.6% were males. The age of patients was ranging between 16 and 29 years, and the average age was 21.7 (95% CI, IQR: 17–25) years. Among migrants surveyed, 89% came from seven Sub-Saharan countries: 20% Gambia, 19% Nigeria, 12% Senegal, 11% Ivory Coast, 10% Ghana, 9% Guinea, and 8% Mali (Figure 2). The remaining 11% come from Burkina Faso, Egypt, Libya, Morocco, Sierra Leone, Tanzania, Tunisia, Chad, Democratic Republic of the Congo, Ethiopia, Kenya, Togo, and Liberia.

In 2014, we evaluated 1001 migrants, 133 tested positive at screening: among them, 117 had LTBI and 16 had active TB (1 intestinal TB). In 2015, we evaluated 688 migrants, 113 tested positive at screening: among them, 99 had LTBI and 14 had active TB. In 2016, we evaluated 944 migrants, 116 tested positive at screening: among them, 101 had LTBI and 15 had active TB. In 2017, we evaluated 1077 migrants, 139 tested positive at screening: among them, 123 had LTBI and 16 had active TB (1 lymphadenitis TB).

**Figure 3:**
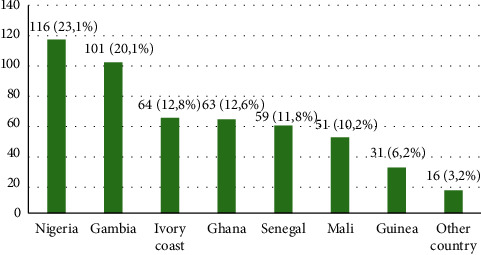
Migrants' country of origin (total TB infection: 501).

We performed 1020 Mantoux tests, among which 131/1020 (12.8%) were positive, and 2690 IGRA tests, among which 370/2690 (13.7%) were positive. Overall, we observed 501/3710 cases of TB infection (13.5%). The TB-infected patients came from Nigeria, 116 (23.1%); Gambia, 101 (20.1%); Ivory Coast, 64 (12.8%); Ghana, 63 (12.6%); Senegal, 59 (11.8%); Mali, 51 (10.2%); Guinea, 31 (6.2%), and other African countries, 16 (3.2%) (Figure 3). The male/female ratio was 4 : 1 (400/101).

**Figure 4:**
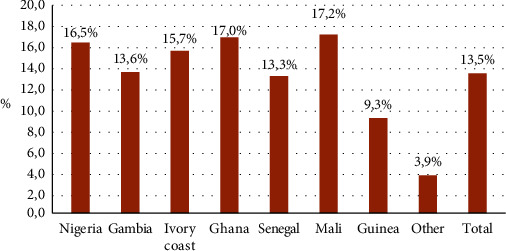
Prevalence of TB infection (total number: 501).

The prevalence of TB infection (LTBI and Active TB) was overall 13,5% (501/3710), Nigeria 16.5% (116/705), Gambia 13.6% (101/742), Ivory Coast 15.7% (64/408), Ghana 17% (63/371), Senegal 13.3% (59/445), Mali 17.2% (51/297), Guinea 9.3% (31/334), and others 3.9% (16/408) (Figure 4).

These percentages are lower than we expected [11].

LTBI was diagnosed in 440 cases (11.8%). 186 (42.3%) migrants lost to follow-up before treatment, 125 males and 61 females. Treatment with isoniazid (300 mg daily) plus rifampin (600 mg daily) for 12 weeks was offered to all the 254 migrants. Treatment completion was obtained in 202 (79.5%) migrants.

Pulmonary active TB was diagnosed in 59 cases (1.6%); extra pulmonary TB was diagnosed in 2 cases (0.1%): 1 TB lymphadenitis and 1 gastrointestinal TB. 11 migrants (18%) lost to follow-up before treatment: 5 males and 6 females. No drug-resistant tuberculosis was observed. All isolates were susceptible to the first-line drugs. Active TB prevalence is lower than other national and international data among migrants [12–16].

Therapies offered to all the 61 cases: isoniazid (300 mg daily), rifampin (600 mg daily), pyrazinamide (1,500 mg daily), and ethambutol (1,200 mg daily) for 2 months, followed by isoniazid and rifampin for another 4 months. TB cure was observed in 45 migrants (90%).

The main reasons why we had such a high loss rate (42.3% in the LTBI group and 18% in the active TB group) prior to treatment are that the migrant escaped from the center, or he/she not want to continue the follow-up, or he/she refused to take medicine.

The reasons why we have an LTBI completion rate of about 80% are as follows: migrants thought they were fine (and did not need any therapy), or did not come to follow-up, or were transferred to another center

## 4. Discussion

In recent years, an unprecedented number of migrants from several African countries have crossed the Mediterranean Sea and reached Italy. According to the New York Declaration for Refugees and Migrants, adopted by Italy among others, countries of transit or arrival should provide basic healthcare to migrant and refugee populations as well as ensure access to basic healthcare services for women and girls (UN 2016). Despite this fact (or nevertheless), the response by healthcare providers and political organizations of the host countries has been uneven across the EU and often inadequate.

Our study confirms that TB infection frequency is high among migrants in Sicily, but lower than that in other Italian and European realities [12–16]. The introduction of successful interventions for both prophylaxis and disease management is feasible.

It is well known that tuberculosis is a disease linked to poverty. Migrants in the precarious and extremely difficult living conditions are forced to experience, during their journey and especially during their permanence in Libya, which represents a loss of health determinants as defined by the World Health Organization, which increases the risk of morbidity [2, 9].

TST and IGRA is suggested by national and international guidelines on migrants and population at risk [9, 10], despite its cost effectiveness being under debate [17].

In our study, the overall prevalence of TB infection was 13.5% (both LTBI and active TB) and the active TB was 1.6%, lower than the prevalence in each country estimated by mathematical modeling [11] (22% for TB infection in the African continent) and lower than other studies among the migrant population in Europe (2% to 5% for active TB) [12, 14, 15].

However, our study found results similar to those conducted at the port of arrival by a questionnaire [13].

The difference in prevalence may be due to some bias in our sample.

About the lower TB infection prevalence, it is important to notice that we have excluded patients needed appropriate care (for example, hospitalization and frequent medical observation by multidisciplinary medical team) and patients escaped from the center. Prevalence in the country of origin was important to consider, but our patients come from a few states of Africa and it does not represent the whole African nations. We could explain the lower active TB prevalence by assuming other similar bias: we observed homogeneous ages of migrants (16–29 years), unlike other studies, and the majority of migrants were in good health condition when leaving their country. Migration represents a stress factor, an important determinant of health, and migrants often cannot access a heathcare service. But, we observe migrants at the port of arrival, unlike other colleagues that observed migrants several months after their arrival in Europe [12, 14, 15], a period of time during which migrants (in heterogeneous age, risk factors, and health status) could experience marginalization, poverty, drug addiction, and crowding, thus increasing the likelihood of infection and disease.

In our experience, the drop-out rate observed was 18% in the active TB and 42.3% in the LTBI, with a total of patients lost to follow-up before treatment of 197/501 (39,3%), too high to the WHO END TB strategy goal. In our opinion, the reasons are to be found in their intrinsic vulnerability. To understand the high drop-out rate reasons, it is important to understand immigrants' views of TB and the obstacles that they face when accessing the health system and adhering to a treatment program, taking into consideration their previous experiences at countries of origin as well as the social, economic, and legislative context when they live at host countries. All this has an important role and should be considered in the design, evaluation, and adaptation of programs.

In this perspective, it is necessary to consider the right to healthcare as an indispensable step, not only for the care of the individual and the community but also to start a process of integration and interaction between the migrant and indigenous population.

In the future, efforts should be made by local authorities to put in place measures to overcome social, economic, and administrative obstacles to the treatment of TB-infected migrants. It will also be beneficial to promote regular training sessions for healthcare providers in order to help them work more effectively with culturally diverse populations.

## Data Availability

Data used in this article are available from the corresponding author upon request.
